# Regulatory Functions of Protein Tyrosine Phosphatase Receptor Type O in Immune Cells

**DOI:** 10.3389/fimmu.2021.783370

**Published:** 2021-11-22

**Authors:** Feiling Xie, Hongmei Dong, Hao Zhang

**Affiliations:** ^1^ Institute of Precision Cancer Medicine and Pathology, School of Medicine, Jinan University, Guangzhou, China; ^2^ Department of Pathology, School of Medicine, Jinan University, Guangzhou, China; ^3^ Department of General Surgery, The First Affiliated Hospital of Jinan University, Jinan University, Guangzhou, China; ^4^ Minister of Education Key Laboratory of Tumor Molecular Biology, Jinan University, Guangzhou, China

**Keywords:** protein tyrosine phosphatases, PTPRO, PTPROt, B cells, T cells, macrophages

## Abstract

The members of the protein tyrosine phosphatase (PTP) family are key regulators in multiple signal transduction pathways and therefore they play important roles in many cellular processes, including immune response. As a member of PTP family, protein tyrosine phosphatase receptor type O (PTPRO) belongs to the R3 receptor-like protein tyrosine phosphatases. The expression of PTPRO isoforms is tissue-specific and the truncated PTPRO (PTPROt) is mainly observed in hematopoietic cells, including B cells, T cells, macrophages and other immune cells. Therefore, PTPROt may play an important role in immune cells by affecting their growth, differentiation, activation and immune responses. In this review, we will focus on the regulatory roles and underlying molecular mechanisms of PTPRO/PTPROt in immune cells, including B cells, T cells, and macrophages.

## Introduction

The protein tyrosine phosphatases (PTPs) catalyze the dephosphorylation of protein tyrosine kinases (PTKs) themselves or their downstream targets and play key regulatory roles in multiple signal transduction pathways (1). Earlier studies have identified a total of 107 genes encoding members of the PTP family in human genome (2). However, the number of the PTP family has increased to 125 recently because some enzymes previously not considered as PTP family have recently been found to possess tyrosine phosphatase activity (3). Based on the amino acid sequence and substrate specificity of each PTP, they have been divided into 4 categories: classical phosphorylated tyrosine (pTyr)-specific PTPs, dual-specific phosphatases (DSPs), Cdc25 phosphatase, and low molecular weight PTPs (LMW-PTPs). Among them, classical pTyr-specific PTPs can be further split into intracellular PTPs and receptor-type PTPs. The former is mainly found in the cytoplasm, while the latter is mainly located on the cell membrane with extracellular domains responsible for ligand-specific binding (4). All PTPs contain a characteristic motif C(X)5R(S/T) in their conserved catalytic domain, rendering them a shared catalytic mechanism (5). Dysfunctional PTPs are responsible for a myriad of human diseases, including cancer, diabetes, autoimmune diseases, and neurological disorders (6).

Proper immune response is essential for a healthy body, while aberrations in immune cell growth, differentiation and activation lead to inappropriate immune response and result in a wide array of diseases (7–9). Although abnormal immune responses are initiated through different mechanisms (10, 11), accumulating evidence suggests that dysfunctional PTPs lead to an imbalance between PTPs and PTKs (12, 13), and subsequently the immune responses. For example, 57-64 PTP-coding genes are generally expressed in immune cells, while 58-76 PTP-encoding genes show specific cellular expression patterns of different lineages (14–16). Protein tyrosine phosphatase receptor type O (PTPRO), also known as glomerular epithelial protein 1 (GLEPP1), PTP-U2, PTP-OC, PTPROt, NPHS6, PTP Phi, belongs to the receptor-type pTyr-specific enzymes, more specifically the R3-subtype receptor-type PTPs (RPTPs), and was first identified in rabbit glomerular epithelial cells (podiocytes) (17). Subsequently, PTPRO has been found in humans, drosophila, mice, and chicken, suggesting that this gene is well conserved among different interspecies (18–20). The PTPRO gene is located on human chromosome 12p12-p13 (18) and is capable of encoding various transcripts controlled by separate promoters in a tissue-specific manner (21). The two most studied isoforms are the full-length PTPRO and the truncated PTPRO (PTPROt), respectively, and both are selectively expressed in distinct cell types. The full-length PTPRO, referred as PTPRO, is predominantly expressed in epitheliums of kidney and brain (18, 19), especially the glomerular epithelial cells in kidney, and therefore is also termed glomerular epithelial protein 1 (GLEPP1). PTPRO comprises an extracellular domain with 8 type III fibronectin-like repeats, a transmembrane domain, and an intracellular catalytic PTP domain (18, 22, 23), and is essential for glomerular filtration (24) as well as synapse formation (25). It has been reported that PTPRO can serve as a tumor suppressor and predictor for diagnosis and prognosis in various cancers (26–30). Besides, downexpression of PTPRO in epithelial cells attributes to promoter methylation in multiple types of cancers, including hepatocellular carcinomas (HCC) (31), breast cancer (28, 32), lung cancer (27) and esophageal cancer (29), suggesting that PTPRO may be a candidate target for tumor epigenetic therapy. Additionally, given the role of PTPRO in restricting tumor-promoting Jak/Stat signaling transduction that is associated with tumor immunity (33), recent studies have also begun to focus on the functional impact of PTPRO on immune cells residing in tumor microenvironment (30, 34, 35).

However, unlike PTPRO, the truncated isoform of PTPRO, known as PTPROt, is produced by an alternative intronic and cell-type specific promoter and is structurally unique with a much shorter extracellular region composed of 8 amino acids (21, 36). Nevertheless, its transmembranal domain and cytosolic catalytic PTP domain are identical to that of PTPRO (36), suggesting that PTPROt also encodes a fully functional protein tyrosine phosphatase that captures and catalyzes specific substrates but may generate disparate effects compared to PTPRO due to different cell types they are in. PTPROt is preferentially expressed in hematopoietic cells and cells of monocyte-macrophage lineage, including osteoclasts (37) and macrophages (38), and is closely associated with osteoclast activity (39) and lymphocyte development (36). The expression levels of PTPRO/PTPROt as well as its substrates in various immune cells are summarized in **Table 1**.

**Table 1:** PTPRO/PTPROt expression, substrates and functions in immune cells.

Immune cell	Expression	Substrates	Functions
B cell	High (36)	Syk (40), Lyn and ZAP70 (41)	Inhibition on B cell proliferation and activation (36, 40);
			promotion on B cell apoptosis (40)
T cell	High (34, 42)	Lck (34)	Promotion on T cell proliferation, proinflammatory factor secretion and T effector cell differentiation (34, 42);
			inhibition on regulatory T cell differentiation (34)
Macrophage	High (38)	Paxillin (43)	Promotion on macrophage motility, chemotaxis, and inhibition on macrophage adhesion (43–45);
			promotion on inflammation and immune response (46–51)
Dendritic cell	High (14, 45)	Undetermined	Undetermined
NKT cell	High (14)	Undetermined	Promotion on inflammation (42)
NK cell	High (42)/Low (14)	Undetermined	Promotion on inflammation (42)
Neutrophil	High/Low (45)	Undetermined	Promotion on chemotaxis (45)
Mast cell	Low (14)	Undetermined	Undetermined

NKT cell, natural killer T cell; NK cell, natural killer cell.

In this minireview, we will summarize the expression, the regulatory roles and the underlying molecular mechanisms of PTPRO/PTPROt in the growth and differentiation of immune cells. We will discuss the PTPRO/PTPROt-regulated immune processes in different types of immune cells and the new insights into PTPRO/PTPROt-related diseases and therapies.

## The Regulatory Functions of PTPRO/PTPROt in B Cells

**Figure 1:**
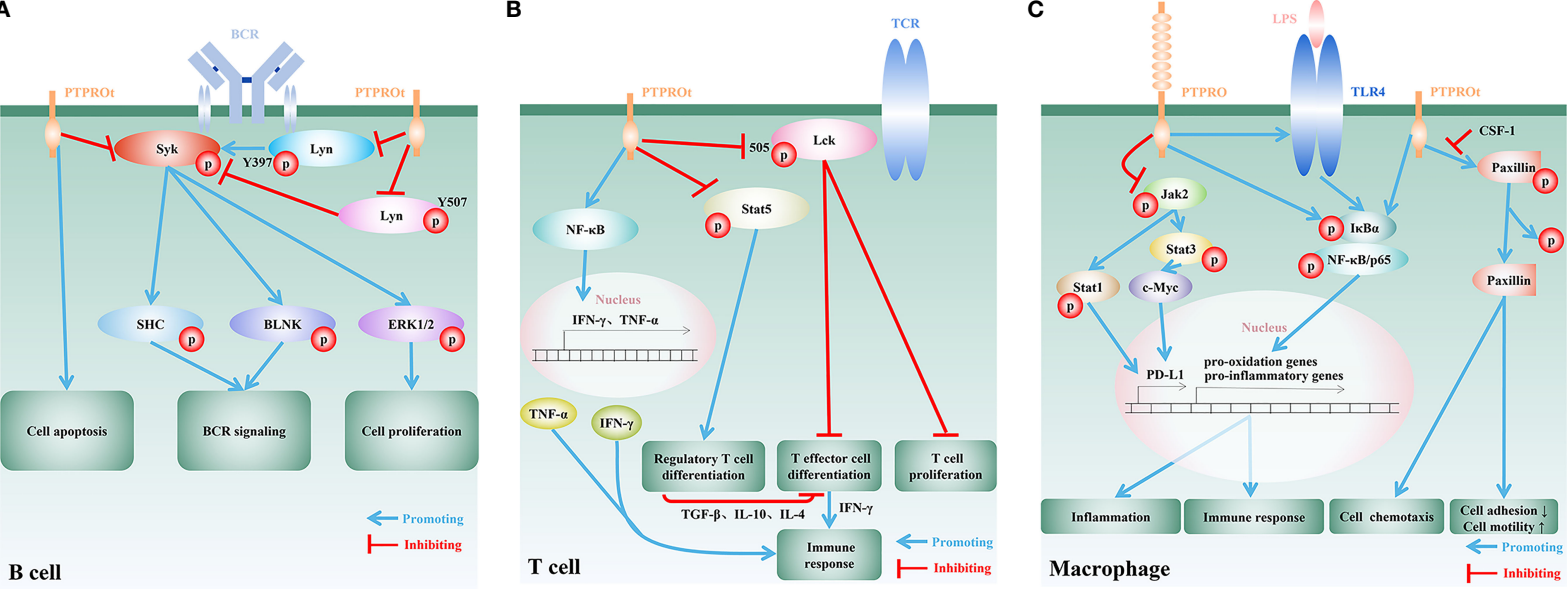
Regulatory functions and mechanisms of PTPRO/PTPROt in immune cells. **(A)** In B cells, PTPROt induces apoptosis and negatively regulates BCR signal cascade amplification *via* dephosphorylating BCR-triggered Syk and Lyn at its active tyrosine phosphorylation site (Y397). Activation of the associated adaptor proteins SHC and BLNK, and downstream signaling events, including MAPK/ERK activation, are blocked by PTPROt, leading to the inhibition of B lymphocyte proliferation. PTPROt can also dephosphorylate Lyn at its inhibitory tyrosine phosphorylation site (Y507) and TCR-associated tyrosine kinase ZAP70 anomalously expressed in human primary chronic lymphocytic leukemia (B-CLL). **(B)** In T cells, PTPROt is mainly responsible for the inhibition of inactive Lck with Y505 phosphorylated, and thus enhances TCR signaling as well as its downstream events, including T cell proliferation and T effector cell differentiation. T effector cells boost immune response *via* the secretion of IFN-γ. PTPROt can also motivate NF-κB signaling pathways, which facilitate the production of pro-inflammatory factors, IFN-γ and TNF-α, and subsequently enhance immunity. On the other hand, PTPROt inhibits the phosphorylation of Stat5 and blocks its promotion on the differentiation of regulatory T cell. The latter negatively regulates immune response by suppressing T effector cell differentiation through secreting inhibitory TGF-β, IL-10 and IL-4. **(C)** PTPROt can promote the motility of macrophages but inhibit adhesion *via* promoting phosphorylated paxillin dephosphorylation, which can be blocked by CSF-1. In an inflammatory environment, such as LPS stimulation, NF-κB signaling pathways are motivated by PTPRO/PTPROt with increased phosphorylated IκBα and p65, which induce the expression of pro-oxidation and pro-inflammatory genes and thus aggravate inflammation and promote immune response. PTPRO can also block Jak2/Stat1 and Jak2/Stat3/c-Myc pathways so as to enhance immunity *via* reducing the expression of PD-L1.

B cell receptor (BCR) is a transmembrane protein located on the surface of B cells and functions as a key regulator of B cell development and adaptive immune response. Like most of the other receptors associated with lymphocyte activation and differentiation, the BCR signaling is largely dependent on the phosphorylation of tyrosine residues mainly by Syk kinases and Src-family kinases (SFKs), such as Lyn (11, 52). PTPROt expressed in B cells was shown to restrain the BCR signaling cascade by suppressing the phosphorylation of Syk and SFKs (40, 41). Another study using a transgenic mouse model with PTPROt overexpressed in B cells, further confirmed the important role of PTPROt in BCR signaling pathway (53). By dephosphorylating the key components of BCR-mediated signaling pathway, Syk and Lyn, PTPROt can promote B cell cycle arrest, induce cell apoptosis, reduce cell proliferation, and is involve in activation and differentiation of B cells (36, 40, 41) (**Figure 1A**). Of note, PTPROt can either activate SFKs by dephosphorylating the inhibitory C-terminal site (Y527/Src, Y507/Lyn) or restrain SFK activity by dephosphorylating its autophosphorylation site (Y416/Src, Y397/Lyn), suggesting that PTPROt can play a dual role in a context-dependent manner in B cells (53). On the other hand, in a mouse model of chronic lymphocytic leukemia (CLL) with endogenous PTPROt complete depletion due to disruption on the distal promoter of PTPRO gene, loss of homozygous PTPROt alleles in CLL cells leads to reduced activity of the BCR signaling, most likely owing to the lack of dephosphorylated Lyn at Y507 (54). Consistent with these data, it has been reported that loss of PTPROt in normal mice shows no significantly abnormal B cell activity, while PTPROt-deficient CLL mice exhibit higher tumor burden and shortened lifespan due to inhibition of the BCR signaling (54). One of the possible explanations for the normal B cell activity in PTPROt-deficient mice is that the function of PTPROt under physiological conditions is redundant for BCR signaling due to other receptor-type PTPs, such as CD45 and CD148 (55, 56). Nevertheless, mouse model with simultaneous absence of PTPROt and other SFK-regulating PTPs in B cells are needed for further confirmation. On the other hand, similar to the non-redundant role of PTPROt in BCR signaling in CLL, downregulation of PTPROt due to frequent methylation has been demonstrated to relate with aggravated diseases evidenced by clinical samples as well as *in vivo* and *in vitro* experiments, which can be attenuated by DNA hypomethylating drug decitabine (5-aza-deoxycytidine) to restore PTPROt expression (41, 57). In this regard, PTPROt seems to be more important in B cell-related lesions. Furthermore, several factors transcriptionally or epigenetically downregulate PTPROt in B cell lymphoma by targeting its promoter directly (58) or indirectly (59). Therefore, a better understanding of the regulatory roles of PTPROt in B cells may offer novel therapeutic strategies in the treatment of B cell-related diseases. In addition, recent studies have emphasized the significant role of B cells in solid tumor microenvironment, aiming to develop novel strategies for tumor immunotherapy and improve the effect and application of immunotherapy (60–62). Tumor-infiltrating B lymphocytes (TIBs) are found in some tumor tissues and are found to be positively correlated with patients**’** response to immunotherapy, offering a new strategy for tumor immunotherapy (63–65). Notably, TIBs respond to BCR stimulation (61). Since PTPROt is involved in B cell development and activation *via* the BCR signaling pathway, it will be interesting to explore how PTPRO/PTPROt of TIBs to reshape tumor microenvironment and effect tumor immunotherapy.

## The Regulatory Functions of PTPRO/PTPROt in T Cells

T cells are the major players of the cellular immunity. Proliferation, differentiation and activation of T cells heavily depend on T cell receptor (TCR)-mediated signaling pathways. SFKs, especially SFK Lck and its downstream ZAP70 are crucial for TCR signaling pathway (66–68). It has been demonstrated that the ectopically expressed Lck and ZAP70 in H293T cells can be dephosphorylated by PTPROt, and that PTPROt-catalyzed dephosphorylation of Lck (Y394) leads to Lck inhibition (41). Although mice with PTPROt deficiency specifically in T cells have not been developed, PTPRO-deficient mice generated by targeting the putative exon 3, the interruption of which also leads to the depletion of PTPROt (24), also provide evidence to unveil the underlying function of PTPROt in T cells. In this respect, *in vitro* study on PTPROt-deficient T cells isolated from PTPRO-deficient mice suggested that Lck as well as TCR are activated by PTPROt (34) **(** **Figure 1B** **)**. Since the level of phosphorylated Lck (Y505) in PTPROt-deficient T cells are higher than that of wild-type T cells, it is proposed that PTPROt promotes T cell proliferation by activating Lck (34). Further investigation found that on the one hand PTPROt promotes T effector cell (Teff) differentiation by activating Lck, and on the other hand it inhibits regulatory T cell (Treg) differentiation by enhancing Stat5 dephosphorylation. These findings suggested that by maintaining the proper Teff/Treg balance in tumor microenvironment PTPROt can enhance T cells’ anti-tumor immune response (34) **(** **Figure 1B** **)**. In addition, in fulminant hepatitis (FH) the PTPROt/NF-κB signaling pathway plays an indispensable role in both innate and adaptive immunity through inducing CD4^+^ and CD8^+^ T cells to secrete IFN-γ and TNF-α (42). Apart from the regulatory role of PTPROt in T cell activation, proliferation and differentiation, a recent study reported the governance of PTPRO on T cell quantity in a novel indirect way through downregulating the expression of PD-L1 on the surface of tumor-associated macrophages (TAMs) (46). The activation of PD-1/PD-L1 signaling pathway is widely involved in a series of processes such as T cell activation, proliferation and apoptosis, and inhibits the cellular immune response mediated by T cells. Tumor cells and tumor microenvironment limit host immune response by upregulating PD-L1 to bind to PD-1 on the surface of tumor-specific CD8^+^ T cells (69). Moreover, the expression of PD-L1 in tumor cells is largely supported by the activation of EGFR, MAPK, PI3K/Akt or Jak/Stat3 pathways (70), some of which are found to be restrained by PTPRO in several cancer types (33, 40, 71). Therefore, it can be implied a novel role of PTPRO/PTPROt as a prohibitor of tumor immune escape, and PTPRO/PTPROt potentially serves as a promising candidate for future therapeutic interventions, shedding a new light on anti-tumor immunotherapy.

## The Regulatory Functions of PTPRO/PTPROt in Macrophages

By serving as the first line of defense against pathogenic microorganisms, macrophages play an important role in both innate and adaptive immunity. Mechanically, macrophages secrete large quantities of cytokines and chemokines, such as IL-1, IL-6, IL-12, TNF-α and CXCL8, to attract other types of immune cells to initiate a local inflammatory cascade (72). Through colony-stimulating factor 1 (CSF-1) and dephosphorylation of paxillin, PTPROt is involved in the regulation of macrophage morphology to decrease adhesion ability, resulting in increased motility and chemotaxis (43–45) **(** **Figure 1C** **)**. Under inflammatory conditions, such as bacterial endotoxin lipopolysaccharide (LPS) stimulation, the macrophages with PTPRO deficiency fail to upregulate both toll-like receptor 4 (TLR4) and TLR4/NF-κB pathway and therefore lead to reduced secretion of pro-inflammatory cytokines. On the other hand, overexpressed PTPRO not only upregulates TLR4 but also promotes macrophage-mediated inflammation, resulting in aggravated local tissue injury and organ dysfunction (47–49) **(** **Figure 1C** **)**. Nevertheless, the precise mechanism underlying the interaction between PTPRO and TLR4 still remains unknown, and mouse model with macrophage-specific PTPRO deficiency could provide a deep understanding of the correlation. Notably, recent study has demonstrated that the inflammation-promoting PTPROt in liver macrophages also forms a negative feedback loop to restrict inflammation by promoting mitophagy to reduce ROS production (73). Therefore, it is of great significance to prevent the destructive effect of PTPRO/PTPROt in macrophages to maintain microenvironment balance. In this respect, several non-coding RNAs have been identified to target to regulate the expression of PTPRO at the posttranscriptional level, offering new targets for the treatment of PTPRO/PTPROt-mediated excessive inflammation in macrophages. For example, miR-6869-5p downregulates PTPRO in placenta-derived mononuclear macrophages and enhances M2 macrophage polarization and thus astricts inflammation (50). MiR-548c-5p acts as anti-inflammatory factor *via* suppressing the expression of PTPRO and the activation of PTPRO/NF-κB pathway in LPS-stimulated macrophages (51). However, more unknown miRNAs have yet to be predicted and proved to regulate the expression of PTPRO in the basic research stage, and *in vivo* experiments are needed to promote clinical transformation. Despite of the pro-inflammation effect of PTPRO, PTPRO in TAMs is found to positively regulate the immune response of T cells and thus suppress tumor progression (46). By prohibiting PD-L1 on the surface of TAMs through blocking Jak2/Stat1 and Jak2/Stat3/c-Myc pathways, TAM-associated PTPRO can prevent T cells from draining in HCC microenvironment (46). This observation raises the possibility that combination of PTPRO and immune checkpoint inhibitors could enhance anti-tumor immunity synergistically.

## The Regulatory Functions of PTPRO/PTPROt in Other Types of Immune Cells

Apart from the above-mentioned immune cells, other types of immune cells, such as dendritic cells (DCs), natural killer (NK) cells, mast cells and neutrophils, are indispensable to the immune system and are displaying promising application values in disease treatment. PTPRO/PTPROt also plays an important role in other immune cells. The analyses on the murine global PTP transcriptome based on the RefDIC database discovered the high expression of PTPRO in immature DCs, equivalent to that of macrophages, while lower level of PTPRO was observed in NK cells and mast cells (14). Although the unchanged mRNA expression level of PTPRO/PTPROt was found in human activated DCs (45) and in BALB/c mouse-derived DCs after 4-hour bacterial LPS stimulation (14), the significant reduction in PTPRO of mouse DCs after 48-hour LPS stimulation suggested that PTPRO may be controlled by subsequent cytokines produced in the early stage but not induced directly by LPS (14). However, whether the unchanged or altered PTPRO/PTPROt expression correlates with DC-associated activities, such as differentiation, proliferation, activation, antigen presentation and so on, remains largely unknown. Based on several PTPs, such as PTEN and SHP-1, confirmed to be negative regulators for DC activation by dephosphorylating and inactivating receptor-associated tyrosine kinases, which affect the antigen uptake and presentation capacity of DCs, it is possible that the membrane-located PTPRO/PTPROt with similar phosphatase activity may participate in the events associated with DC activation (74). In addition, PTPRO-deficient hepatitis mouse model induced by Con A, a hepatic inflammation inducer, was constructed to investigate the activation and function of NK/NKT cells (42). This *in vivo* investigation on the inflammatory PTPROt-deficient NK/NKT cells isolated form PTPRO-deficient mouse spleen and liver mononuclear cells using magnetic beads demonstrated weakened activation and damaged function of NK/NKT cells with less detectable IFN-γ and TNF-α, which is possibly attributed to the NF-κB signaling inactivation (42). These data preliminarily indicated that PTPRO/PTPROt is also an important factor for the maintenance of NK/NKT cell function. Furthermore, since the PTPRO/PTPROt catalytic domain encoding mRNA expression is readily detectable in mouse neutrophils, inhibitors that targeted to PTPRO/PTPROt prohibit thioglycolate-induced peritoneal chemotaxis of neutrophils probably through blocking PTPRO/PTPROt-mediated dephosphorylation of certain substrates that are essential for neutrophil motility (45), like the above-mentioned paxillin in macrophages. However, mechanistic investigation on how PTPRO/PTPROt regulates these subsets of immune cells remains largely to be explored. Therefore, more functional studies are badly needed to illustrate the exact role of PTPRO/PTPROt in these immune cells in the future so as to achieve more therapeutic effects.

## Discussion

In addition to a potent tumor suppressor, PTPRO/PTPROt also plays a crucial role in the signaling pathways in B cells, T cells and macrophages. PTPROt has dual impact on B cells *via* dephosphorylating a key member of the BCR signaling pathway, Lyn at its active and inhibitory tyrosine residues under different circumstances, albeit it is mainly responsible for the deceleration of B cell-related cancer progression. PTPRO/PTPROt is also capable of positively enhancing anti-tumor immunity in both T cells and macrophages while brings about negative effects in several macrophage-related inflammatory diseases. Therefore, PTPRO/PTPROt confers a promising therapeutic target in inflammation and cancers and other relevant diseases. However, with the development of advanced technologies, such as RNA sequencing, proteomics, CRISPR and lineage tracing, more undiscovered and cell-specific substrates and upstream regulators of PTPRO/PTPROt remain to be explored to unveil the comprehensive network of PTPRO/PTPROt. Given the inadequate understanding of the roles of PTPRO/PTPROt and the underlying mechanisms in other types of immune cells, additional studies are needed to resolve the unsettled issues proposed in this review.

## Author Contributions

HZ and HD discussed the structure and organization of the manuscript. FX and HD wrote the manuscript. HZ supervised and revised the manuscript. All authors contributed to the article and approved the submitted version.

## Funding

The work was supported by a grant in part by the National Natural Science Foundation of China (82072683, 81773087, 81071736, 81572876 and 30973508 to HZ, 81802404 to HD); Natural Science Foundation of Guangdong Province of China (2021A1515012522 and 9151018004000000 to HZ); Science and Technology Planning Project of Guangdong Province of China (2019A030317024 to HZ); Natural Science Foundation of Guangdong Province, China (2021A1515012522 to HZ, 2021A1515011028 to HD); Special Project on the Integration of Industry, Education and Research of Guangdong Province (2011A090100024 to HZ); Jinan university Innovation and Entrepreneurship Fund for College Students (202010559081 and 202110559097 to HZ).

## Conflict of Interest

The authors declare that the research was conducted in the absence of any commercial or financial relationships that could be construed as a potential conflict of interest.

## Publisher’s Note

All claims expressed in this article are solely those of the authors and do not necessarily represent those of their affiliated organizations, or those of the publisher, the editors and the reviewers. Any product that may be evaluated in this article, or claim that may be made by its manufacturer, is not guaranteed or endorsed by the publisher.
